# Computer vision meets microfluidics: a label-free method for high-throughput cell analysis

**DOI:** 10.1038/s41378-023-00562-8

**Published:** 2023-09-21

**Authors:** Shizheng Zhou, Bingbing Chen, Edgar S. Fu, Hong Yan

**Affiliations:** 1 State Key Laboratory of Marine Resource Utilization in South China Sea, Hainan University, Haikou, 570228 China; 2 Graduate School of Computing and Information Science, University of Pittsburgh, Pittsburgh, PA 15260 USA

**Keywords:** Optical sensors, Electrical and electronic engineering

## Abstract

In this paper, we review the integration of microfluidic chips and computer vision, which has great potential to advance research in the life sciences and biology, particularly in the analysis of cell imaging data. Microfluidic chips enable the generation of large amounts of visual data at the single-cell level, while computer vision techniques can rapidly process and analyze these data to extract valuable information about cellular health and function. One of the key advantages of this integrative approach is that it allows for noninvasive and low-damage cellular characterization, which is important for studying delicate or fragile microbial cells. The use of microfluidic chips provides a highly controlled environment for cell growth and manipulation, minimizes experimental variability and improves the accuracy of data analysis. Computer vision can be used to recognize and analyze target species within heterogeneous microbial populations, which is important for understanding the physiological status of cells in complex biological systems. As hardware and artificial intelligence algorithms continue to improve, computer vision is expected to become an increasingly powerful tool for in situ cell analysis. The use of microelectromechanical devices in combination with microfluidic chips and computer vision could enable the development of label-free, automatic, low-cost, and fast cellular information recognition and the high-throughput analysis of cellular responses to different compounds, for broad applications in fields such as drug discovery, diagnostics, and personalized medicine.

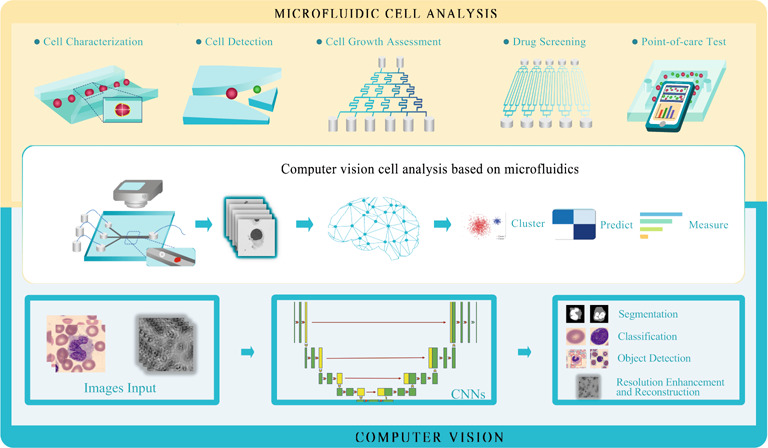

## Introduction

With the rapid advancement of interdisciplinary research in micromanufacturing, microelectromechanical system (MEMS) and life science, the fabrication of microfluidic devices has increasingly attracted international attention in the biotechnological field. Various cell detection devices, such as electrodes, optics and acoustics, can be integrated on a microfluidic chip, and the structure and function of the chip can be flexibly designed^1^, which can not only meet the needs of various types of cell analyses but also enable the visualization of single-cell information. Moreover, a series of operating steps, including sample preparation, reaction, separation, detection, cell culture, sorting, lysis and so on, which are available in chemical and biological labs, are miniaturized into one microfluidic chip to achieve high-throughput and high-speed detection.

Cell analysis is a complicated process that involves various cell types and multiple biological reactions through different temporal and spatial dimensions^2^. Even in the same physical environments and culture conditions and with the same external stimuli, microbial cells from the same metrocyte may have remarkable variation in terms of size, growth rate, morphology and phenotype. Traditional cell assay methods, such as enzyme-linked immunosorbent assay (ELISA)^3^, quantitative polymerase chain reaction (qPCR)^4^ and other common bioassays, can provide unique insights into molecular biology and cell biology. However, these methods seem to neglect important cell morphology and cell state information and are thus unable to elucidate biological phenomena or accidental events caused by cellular interactions. Therefore, the analysis of cell composition, structure and morphology at the single-cell level is of critical importance, not only for the exploration of cell heterogeneity but also for the discovery of interactions among cells and between cells and the environment. It enables us to comprehensively understand the behavior of cells, tissues, organs and even whole life forms.

Optical imaging has been the most direct way to observe microbes at the single-cell level since the invention of microscopes by Antony van Leeuwenhoek in the mid-17th century. Currently, microscopic observation has become a routine procedure for biological scientists and bioengineers to examine cell images and determine their viability. Common imaging methods include optical bright field microscopy, fluorescence microscopy, confocal laser scanning microscopy, super resolution imaging, lensless microscopy and so on. Cellular or subcellular features are associated with characteristic structures and functions of living systems and play an important role in cell identity^5–8^. Cell images obtained through microscopic imaging contain a large amount of cellular information, such as size, morphology, texture, internal structure. As opposed to the traditional single variable cell analysis method, the main advantage of image-based cell analysis lies in its ability to implement high-throughput screening for single-cell and multivariable imaging analysis. For example, in drug screening, visual cellular data can be interpreted to determine cell phenotypes, drug-induced gene expression and protein localization, to predict phenotypic changes in cytoskeleton structure and to implement high-throughput screening for variable single-cell imaging^6,9^.

**Fig. 1:**
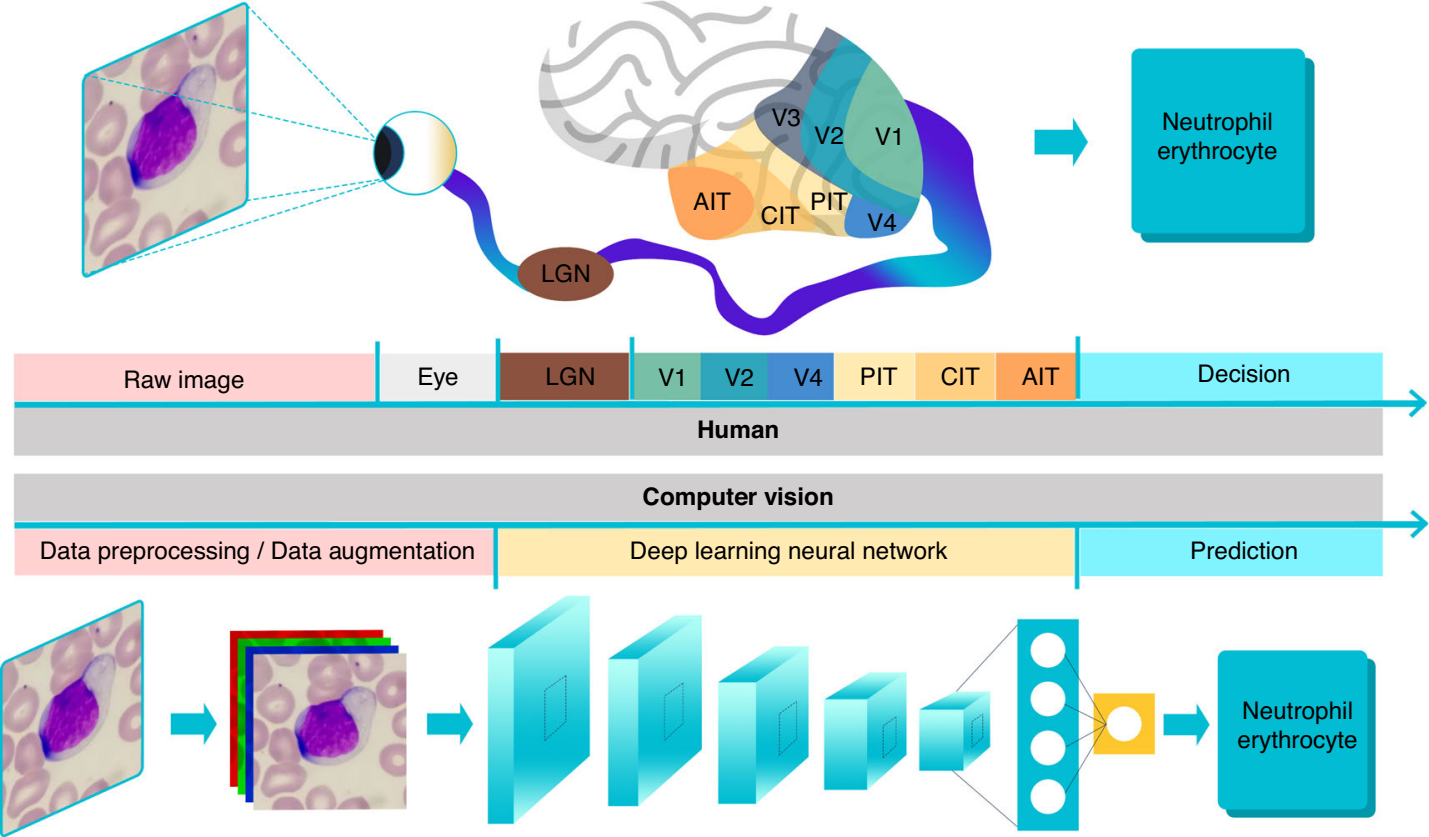
Cell recognition through the human eyes and brain and computer vision. PIT: posterior IT, CIT: central IT, AIT: anterior IT

The development of imaging technology has led to image quality enhancement that enables more information, including subtle phenotypic changes of cells that are difficult to recognize by the human eye, to be obtained from individual images. At the single-cell level, images may contain a large amount of cell morphological and spatial information. However, only a small part of this information can be extracted from cell images using manual analysis due to the large amount of data. Thus, there is an urgent need for the development of hardware and algorithms to provide a real-time data mining and visualization platform for computerized cell visual analysis. The overwhelming information content and the growing data volume call for automated image analysis methods with upgraded information content. In recent years, computer vision algorithms have been widely used in biological image processing and analysis to overcome the bottleneck caused by the explosion of data growth since they are able to extract and analyze massive visual information from cell images^10–12^. Computer vision is a branch of artificial intelligence (AI) that uses algorithms to mimic the way the that the human visual system acquires, processes, analyzes and synthesizes visual information. The human visual cortex is composed of over 140 million neurons and is capable of unrivaled visual perception by segmentation and integration of visual data relayed from the retina, as well as by instantaneous recognition of objects and patterns (Fig. 1). Light signals are converted into electrical signals on the retina and transmitted to the lateral geniculate nucleus (LGN). This process is similar to the image preprocessing and image enhancement processes in computer vision, which facilitates the recognition and extraction of targeted object features in the image. The LGN then transmits the signals to the visual cortex (including V1, V2 and V4) and the inferior temporal (IT) cortex to further refine the signals, extract the color, shape and movement information, and make conclusions. This process is similar to the image recognition process of a deep learning neural network, where the neural network extracts the feature information layer by layer and finally outputs the classification probability of each category. For the five senses that the human brain possesses, vision plays a major role that involves >50% of the brain’s computational space directly or indirectly for constant visual processing and integration. Humans use more neurons for vision than the other four types combined. This is the reason why human vision is so powerful. In comparison, computer vision is still in an immature stage of development with respect to visual perception.

Although AI-based image recognition is not yet matching the human and computer vision still lags behind the human visual cortex thus far, we argue that when computer vision algorithms are integrated with microfluidic technology in cell data acquisition, the integration of computer vision with miniaturized microfluidic devices is somewhat superior in cell imaging analysis to human intelligence. The reason is that even though image recognition in the visual cortex is more advanced than its artificial counterparts, the human eye is the bottleneck for information flow from microscopic objects to the retina. On the other hand, microfluidic chips for cells are capable of generating much more information intense visual data at the micrometer and even nanometer level for subsequent computer vision equipped with artificial neural networks to learn, analyze and recognize visual information.

This review will be conducted with the abovementioned theme to show that the combination of computer vision and microfluidics will not only promote the real-time detection of single cells but also enable more accurate and robust processing of high-throughput visual data. Computer vision algorithms can precisely overcome the analytic difficulty of microfluidic technology and make the analytic results more accurate and robust, providing a promising AI-aided cell visual data generation and information acquisition method in the fields of life science and biotechnology. This review will discuss the principles and related applications of computer vision and microfluidics and provide an outlook on the future development of this interdisciplinary research.

## Computer vision for cell analysis

**Fig. 2:**
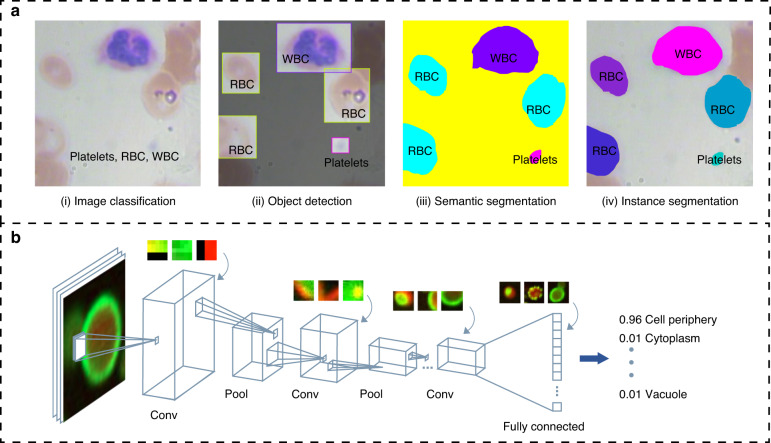
Computer vision for image analysis. **a** Four major applications in the field of computer vision. (i) input an image and determine the class to which the image belongs; (ii) detect all objects in an image, determine their classes and locate the positions of the objects; (iii) on the basis of object detection, semantic segmentation requires further determination of which pixels belong to a class in an image; (iv) determine which pixels each object contains on the basis of object detection; **b** Convolutional neural network for cell feature extraction and classification. CNN can detect the features of corners, edges, and lines in the first layers, and its middle layer represents a combination of these low-level features, while at deeper levels, there are features that give the most weight to the cells that need to be separated, such as the membrane structure and dot patterns^120,121^

There are four basic tasks in the field of computer vision, including classification, location, detection and segmentation, as shown in Fig. 2a. Since an image is a matrix composed of pixel values, traditional computer vision methods can extract features from an image to distinguished it from other classes of images, such as size, deformation, brightness, edge, texture and color, allowing the computer “understand” the image. These features are expressed in the form of numerical values, vectors, and symbols. Algorithm optimization and feature engineering for specific cells is a challenging task. Tuned feature extraction algorithms have a small amount of computation and fast processing speed and can handle high-throughput and relatively simple image processing tasks. However, they are not sufficient in terms of addressing global feature changes and local context changes. In recent years, with the improvement of computing power and artificial intelligence algorithms, neural networks have been widely used in practice. A neural network consists of several neurons that are divided into several layers, including an input layer, a hidden layer, and an output layer for classification or prediction.

To make neural networks more suitable for image analysis, researchers proposed convolutional neural networks (CNNs)^13,14^, and the architecture of CNNs was inspired by the visual data processing mechanism underlying the human visual cortex. Convolutional neural networks use convolutional kernels to process local image information in the form of a matrix and are the most mainstream neural network in the field of computer vision. Convolutional neural networks are composed of convolutional layers containing multiple convolutional cores. It can directly learn the features at the pixel level of a cellular images. The receptive field of the front convolutional layer is small, and most of the captured and calculated images are local information with picture details, while the back convolutional layer increases layer by layer to capture more complex and abstract information of the image (Fig. 2b). Convolutional neural networks identify objects through their ability to extract more abstract features, and can capture more variations and details of visual stimuli and perform more complex cell image analysis tasks.

## Exploration of computer vision in microfluidic cell analysis

Currently, the development of microfluidic technology is at a pace where it produces more data than researchers are able to analyze, especially in the cell imaging area using microfluidic cell analysis. Computer vision algorithms can precisely solve this bottleneck with the aid of microfluidic technology. With their excellent feature extraction and generalizability, computer vision algorithms have been widely used in the development of cell characterization in microfluidic cytometry, cell detection on chips, cell growth analysis, drug screening and point-of-care tests.

### Cell characterization in microfluidic cytometry

The biophysical features of cells reflect their identities, which are fundamental to maintain their homeostatic state in health and play a defining role in the pathogenesis of disease^15^. Biophysical cytometry is a technique used to analyze the physical features of cells, including their size, shape, deformability, and mechanical properties^16^. It can reveal subtle spatial and temporal variations in the biophysical phenotype of cells and their correlation with cellular function.

**Fig. 3:**
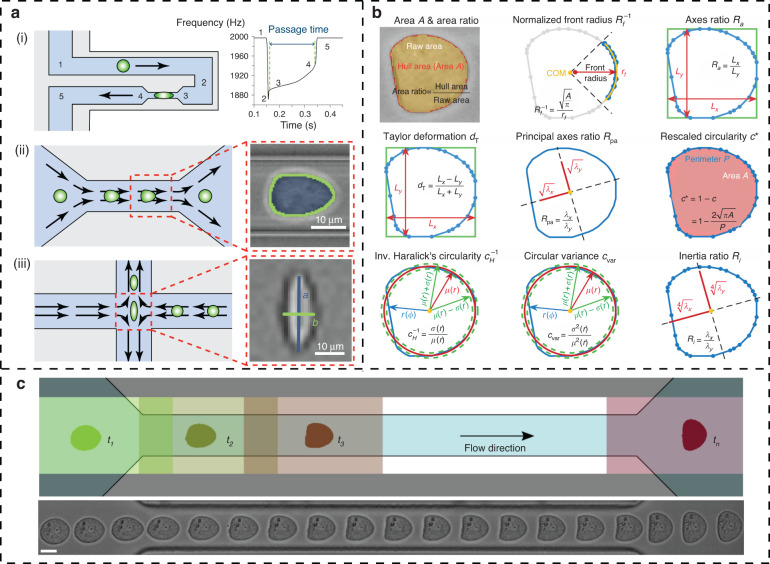
Traditional computer vision algorithm-assisted microfluidic cytometry. **a** The three main types of deformability cytometry^23^. (i) cDC measured the time needed for cells to pass through the constricted channel; (ii) sDC and (iii) xDC use hydrodynamic flow to induce cell deformation without direct contact and infer cell deformability from the image-based evaluation of cell shape. **b** Nine common shape descriptors^27^. They are used to measure the degree of deformation of cells after being focused by the buffer. Different features are selected to construct a scatter plot that shows the ranges of features of different cell classes. **c** Dynamic tracking of single cells in a microfluidic channel^28^. Multiple ROIs are set up in the channels to collect time series images of cellular deformation

Traditional computer vision techniques have been used in cell characterization for several decades^17^, and they remain a valuable tool for analyzing cell images, offering advantages such as flexibility, interpretability and computational efficiency. Traditional computer vision algorithm-assisted label-free microfluidic imaging cytometry and deformability cytometry can achieve extremely high cell throughput^18–20^. Microfluidic chips offer a microfabricated detection region with fine-tuned geometrical properties^21^ and ensure that only a single cell is present in the field of view at any given time by enabling the precise control and manipulation of fluids at the microscale level^22,23^. This allows for the acquisition of high-quality images of individual cells, and for the extraction of detailed information of spatial and temporal features. Deformability cytometry enables high-throughput detection of both static and dynamic cellular mechanical phenotypes and has been widely applied in various fields, including cancer research, stem cell differentiation^18^, and blood testing^24–26^. Currently, constriction-based deformability cytometry (cDC), shear flow deformability cytometry (sDC) and extensional flow deformability cytometry (xDC) are mainly used^23^, as shown in Fig. 3a.

When applying traditional computer vision methods to microfluidic imaging cytometry, the general steps are image acquisition, preprocessing, segmentation, feature extraction and analysis. Many of these processes can be implemented through the computer vision library OpenCV (https://opencv.org/). The most popular method in imaging flow cytometry is to convert a single-cell image into a binary map and then perform threshold segmentation to distinguish the cell from the background and extract spatially-based biophysical features by means of built-in functions or fine-tuned feature engineering, including cell size, perimeter, circularity, area, deformation, uniformity, density and so on, as shown in Fig. 3b. The above steps can be carried out in turn with the OpenCV functions ‘*threshold*’ and ‘*findContours*’. In addition, the ‘*moment*’ and ‘*HuMoment*’ functions can be used to extract the moments and Hu moments, respectively, to calculate features such as the centroid and orientation and for more comprehensive shape recognition.

The temporal deformation features of cells can be realized by setting multiple ROIs in a channel for continuous cell tracking. Fregin et al. captured the full dynamics of suspended cells passing a centralized construction of a microfluidic channel on-the-fly by sDC, determining the apparent Young’s modulus and apparent viscosity with throughput rates of up to 100 cells per second^27,28^, as shown in Fig. 3c. The integrability of microfluidics allows the integration of other physical fields on a chip to stimulate cells for short-time transient responses. Although this research has not yet achieved high throughput, the technique is promising in terms of uncovering physiological responses of single cells by characterizing the temporal and spatial biophysical features in real time and tracking cell cycle stages.

Deep learning offers the potential to extract hidden information from images captured by imaging flow cytometry^29^. Unlike traditional computer vision methods, deep learning methods do not require to predefine the morphological features to be included in the analysis and do not search for the optimal combination of features in a feature set. It will avoid biased analysis, allow the exploration of potential relationships between different phenotypes and aid in the discovery of the heterogeneity of cell types^15,30,31^. Although achieving detection throughput comparable to that of traditional image processing methods is not yet possible through the combination of deep learning methods and microfluidic cytometry, this approach is still very promising, especially in terms of obtaining a deeper understanding of biophysical cellular heterogeneity and revealing significant biophysical biomarkers of health and disease.

### Cell detection and classification on a chip

Different from external physical fields such as sound, electricity and magnetism, the optical imaging system on a microfluidic platform can intuitively obtain the internal structure and phenotypic features of each cell with almost no damage to the cells. The characteristics of miniaturized microfluidic chips with high integration make them an ideal platform to automatically analyze cell image or video data combined with computer vision-related technologies, providing a tool to automatically analyze cell image or video data combined with computer vision methods^32^.

The integration of computer vision and microfluidic chips makes it possible to realize cell detection, cell identification and cell sorting on a chip. Before sorting, it is necessary to fully understand the features of each cell, which can be adequately learned from previous cell imaging data. After extracting the cell characteristics, the software transmits the sorting signal to a sorting device to implement the sorting process. Cell image segmentation methods based on feature engineering mainly include threshold segmentation^33,34^, watershed algorithms^35–37^, and edge detection^38–40^. Unlike those of the single-cell images generated by imaging flow cytometry with a uniform background, general image classification tasks have a high level of background noise and multiple objects, which makes traditional algorithms require time-consuming tuning processes, and they are less robust to environmental perturbations.

The advantage of using CNNs for cell classification is that they enable feature extraction and encapsulation without requiring user to concern about the specific feature treatment process, and their portability has allowed them to be widely used in the field of microfluidic cell classification. Nitta et al. proposed an Intelligent Image-activated Cell Sorting system (iIACS) based on a CNN architecture that is capable of sorting cells according to cell images in real time. The system integrates a high-speed fluorescence microscope, a 3D two-step hydraulic focus microfluidic chip, and the dual-membrane pumps to enable real-time and automated fluid focusing, cell detection, and cell sorting. It allows the real-time sorting of microalgae and blood cells based on intracellular protein localization and cell‒cell interactions^41,42^. Based on iIACS, the authors changed the cellular imaging method to an image-sensor-based optomechanical flow imaging method and upgraded the system hardware to obtain higher quality cell images and faster processing speed^43^. In another case, Chen et al. developed an optomechanical scanning imaging m ethod to quickly transmit cell signals, reconstruct cell images through a field-programmable gate array (FPGA), and transfer the reconstructed cell image to the CPU for image feature extraction and cell classification^44^. The study by Godino et al. demonstrated a precise single-cell manipulation technique in which an automated cell sorting device was fabricated using an open-source package in the field of computer vision to recognize cell color and manipulate cell movement through dielectrophoresis under bright field imaging^45^. Nawaz et al. used surface acoustic waves to manipulate cells and accurately sort single cells based on the output of deep neural networks^46^. Girault et al. developed a label-free microfluidic droplet sorting system based on droplet images. In their system, a high-speed camera was used to capture the cells in the droplets in each frame and recognize their boundaries. Then, computer vision was used to recognize the cell images based on the morphological characteristics of the objects in the droplets and to sort the droplets of interest through direct current pulses^47,48^.

**Fig. 4:**
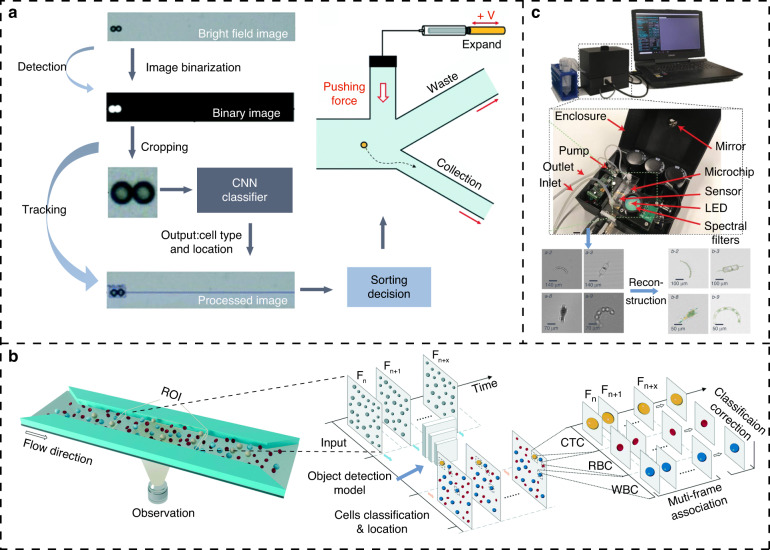
On-chip cell detection and classification with computer vision technologies. **a** CV-based image-activated microfluidic cell sorting^50^. Image segmentation algorithms and object detection neural networks in computer vision were applied to quickly collect cell images in a high-throughput manner and output the sorting decision to the downstream piezoelectric actuator. **b** The YOLOv4 algorithm with multiframe image correlation to detect CTCs in a sheathless and label-free manner. Multiple frames of a cell in the flow channel can reflect the phenotype of that cell from multiple perspectives, and together, these images are used to make classification decisions, greatly improving the accuracy of cell classification as well as the robustness of the model. **c** Lensless portable flow cytometer for natural water analysis^62^. This device uses a partially coherent lensless holographic microscope to capture images with diffraction patterns of flowing objects in microchannels and then converts these diffraction patterns into chromatic variations for a deep learning neural network to be trained for the classification and identification of algae

Cell detection and counting with microfluidic chips is characterized by fast and high throughput and the large-scale imaging of cells in chip channels; in particular, cells in the field of view are detected and counted automatically in real time by computer vision technology. Heo et al. proposed a fast image-processing pipeline (R-MOD: Real-time Moving Object Detector) based on CNNs to acquire and analyze cell images through monitoring microfluidic channels real-time^49^. They also added a syringe-connected piezo actuator to enable rapid cell sorting after detecting and counting cells^50^, as shown in Fig. 4a. Here, computer vision and microfluidics were applied to detect multiple cells in the field of view and to maintain high-throughput detection by increasing the concentration of the cell solution at a low flow rate. Transferring algorithms from the field of pattern recognition to microfluidic cell detection is promising. For example, the You Only Look Once (YOLO) series of algorithms^51–53^, which are lightweight, modular and fast, have been used for droplet^54,55^, cellular viability^56^ and circulating tumor cells (CTCs) detection^57^. It can also be integrated with other algorithms to achieve different assay functions, as shown in Fig. 4b. The dynamic processes of a cell are recorded in multiframe images, which is important for rare cell characterization. It should be noted that real-time detection using computer vision described above did not involve additional channels or syringe pumps to focus the cell solution because the cells flowing through the channel without a serial arrangement were well detected and identified by the object detection algorithm in computer vision.

The performance of the image sensor of the microscope camera determines the resolution of the collected cell images. Some cell substructures need to be displayed as high-resolution images. The lensless microscope imaging method is not bound by the microscope lens and can achieve high-resolution imaging in a large field of view^58^. It acquires the digital signals of cell samples through the sensor and reconstructs them into a high-resolution image through a CNN to realize the rapid detection of the cells^59–61^. In recent years, some researchers have applied lensless microscopic imaging technology to microfluidic cell analysis. Gӧrӧcs et al. developed a real-time water detection device to display the flow of the diffraction patterns of microorganisms in microfluidic channels through lensless holographic microscopy. A holographic diffraction pattern was established in real time by using the phase recovery and image reconstruction method in computer vision, and chromatic image of individual microorganisms were captured. The method is low cost and portable and achieves high-throughput continuous monitoring of marine microorganism groups; for example, it can obtain the composition of phytoplankton in the ocean^62^, as shown in Fig. 4c. Singh et al. developed a microfluidic system for online digital holographic microscopic imaging that was able to detect and identify CTCs in the blood at a rate of 10,000 cells per second without cellular labeling^63^. Luo et al. combined mobile lensless microscopy with deep learning to automatically measure levels of highly sensitive C-reactive protein in human serum samples^64^. The lensless microfluidic system can quickly obtain high-quality single-cell images without limiting the throughput of the system, providing an economical, effective and portable solution for real-time diagnosis.

The massive imaging datasets generated by microfluidic chips meet the demands of neural networks that rely on large amounts of training data for accuracy. The “big data” feature and the ability to sort cells with microfluidics based on image features marks an important milestone for the integration of computer vision and microfluidic chips as an important tool for enabling AI-based research in life science and biomedicine. For example, biomedical research can rely on massive image features from microfluidics to understand cell behaviors and find their relations between phenotype and genotype. With algorithm and computing power updates, the image detection and feature extraction abilities of computer vision will make its applications in microfluidic cell analysis more intelligent.

### Cell growth assessment on a chip

Cell growth assessment is an important task in biological research to obtain a deeper understanding of cellular activities such as the cell life cycle, cell growth, metabolism and cell population communication. It includes the assessment of cell viability, the living or dead state of cells in the cell community, the cell growth cycle, and the possibility of cell survival under environmental stress. Compared with that of the entire microbial population, the assessment of cell growth status at the individual cell level can provide more detailed information^65^.

Microfluidics are fast, efficient and economically effective as a cell growth detection platform. They can detect the status of cell metabolism to observe the cell phenotype at the single-cell level and to create a chemical and physical microenvironment for cells by fully manipulating the fluid inside and out. A microfluidic device can also allow cell culture experiments with different concentration gradients or pH gradients through solution exchange in the microchannel. Whether at the single-cell level or in the cell population, cell image analysis through computer vision can intuitively detect cell morphological changes during cell growth or environmental variation with much lower costs^66^ compared to that of electrochemical detection and other methods. Jagannadh et al. demonstrated a portable bright-field imaging system based on a microfluidic chip to detect the cell viability of yeast cells^67^. Even using low frame rate imaging equipment, computer vision algorithms were able to automatically segment the cell images in real time, extract cell features, and evaluate the effects of the elimination, absorption and retention of dyes. They were even able to quantify the dead cells from a large number of viable cells. Kim et al. proposed a new computer-vision-based method to estimate the viability of bacteria in a microfluidic channel using fast Fourier transform to detect the frequency alteration of a microscopic image^68^. By analyzing the frequency of time-lapse images, the regional concentration changes of bacteria cultured under an antibiotic gradient were detected. Chen et al. used CNNs to automatically collect and segment tumor cell images of cell cultures on a microfluidic chip^69^. By associating the cell shapes in the early cell growth phase with those in the final phase, the CNN model was able to predict whether a cell would eventually form a tumor sphere from the cell growth images from the fourth day, which greatly reduced the time required for medical oncology experiments. It was also observed that computer vision with microfluidics can be used to assess the degree of cell division cycle and cell differentiation, which has been mentioned in more than one study^70,71^.

### Drug screening on a chip

**Fig. 5:**
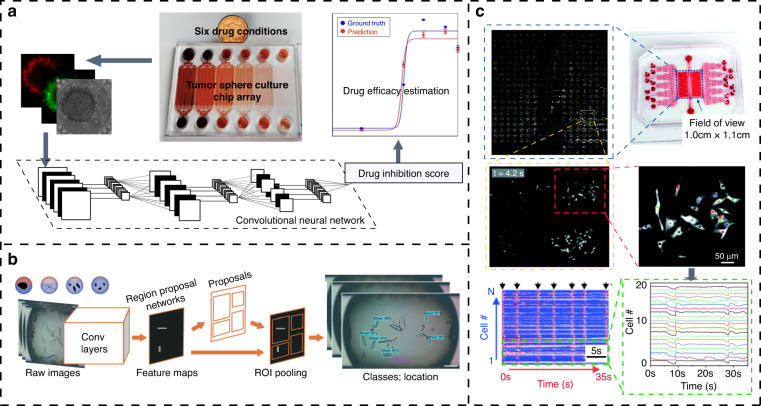
CV-based drug screening system. **a** Prediction of the therapeutic effect of drugs on tumor spheres by a convolutional neural network^74^. The chip can simultaneously test six drug conditions and capture bright-field images and live/dead cell staining images of tumor cells after drug treatment. The bright-field images are input into a convolutional neural network to predict the viability of cells, and the living/dead cell staining images are used as the true values to evaluate the accuracy of the prediction results. **b** Combination of a hydrogel droplet platform and computer vision to screen the antisolvent crystallization conditions of active pharmaceutical ingredients^75^. The method collects images of hydrogel droplets containing different drug crystals in serpentine channels and detects different drug crystal shapes in the hydrogel droplets by using an object detection algorithm. **c** Real-time drug screening by ultralarge-scale high-resolution imaging and computer vision. Video clips of Ca2+ ion signals in cells are recorded at 30 Hz and then analyzed offline. Within each region of interest, all image frames are accumulated to synthesize a grayscale map, and individual cells are then identified by the binarization algorithm ImageJ. The fluorescence intensity of these cells in each frame are extracted and resolved, revealing the rhythmic response of cardiomyocytes following drug injection^78^

Unlike traditional well plate cell culture methods, microfluidics provide the ability to manipulate cells and maintain a controlled environment for cell growth in a three-dimensional environment. The thousands of microchambers on a chip allow high-throughput drug testing and screening for biomedical investigations. Although there are many applications for drug screening through microfluidic platforms^6,72^, progress has been slow in large-scale drug screening. One of the main bottlenecks is the lack of a fast and low-cost read-out method to screen drugs. In recent years, the emergence of computer vision methods has gradually overcome this obstacle. Drug screening based on cell phenotype has become a trend in drug discovery, which is an unbiased screening to predict and discover compounds that induce cell phenotype changes in a cell growth environment. Research shows that these drug screening methods have discovered more new drugs than targeted drug screening based on specific targets or mechanisms^73^. Zhang et al. developed a microfluidic platform that can culture three-dimensional tumor cells for cell growth assessment and drug screening (Fig. 5a). This platform can perform automatic image acquisition and cropping^74^. By training bright-field images of three-dimensional tumor cells, a CNN model predicted and estimated the viability of 3D tumor cell spheroids and accurately evaluated the efficacy of three chemotherapeutic drugs.

The crystallization screening of pharmaceutical active ingredients is another important drug discovery approach in the pharmaceutical industry, where microfluidic technology provides a new method for the high-throughput screening of pharmaceutical crystallization. Although it has the advantages of low sample consumption, less time consumption, and increased throughput, there are still some problems that need to be solved. For example, extracting useful information from a large amount of data requires long-term and tedious labor. To overcome these hurdles, Su et al. proposed a high-throughput system that combines microfluidic hydrogel droplet platforms with computer vision to screen drug crystallization conditions (Fig. 5b). This system uses a CNN to identify and classify hundreds of indomethacin crystal images and successfully determines the antisolvent crystallization conditions for three indomethacin crystals^75^.

The abuse of antibiotics has promoted the evolution of drug-resistant bacteria. To address this threat, the development of novel and specific antibiotics is crucial and requires timely antibiotic sensitivity testing. The drug susceptibility testing microfluidic (DSTM) system developed by Matsumoto et al. used image analysis software to detect the difference in the number and shape of drug-treated cells. It was found that this method was able to quickly detect the drug sensitivity of Pseudomonas aeruginosa^76^. Yu et al. performed real-time imaging of freely moving bacterial cells in urine and used deep learning algorithms to automatically analyze various phenotypic features and responses of the cells in videos. By learning multiple phenotypic features of cells, researchers can determine whether bacterial cells are inhibited by antibiotics without the need to define and quantify each feature^77^. It is obvious that computer vision neural networks have great potential in drug screening since high-throughput microfluidic systems are able to define and quantify every feature of the cells in massive 3D cell image acquisition for the CV neural networks to use as training data.

It is possible to obtain a variety of phenotypic features of individual cells, including cell size, morphology, movement and division, using computer vision methods to analyze cell images under the action of drugs. Cells dynamically grow, change shape, divide, rotate and move over time. Using conventional computer vision techniques by defining and quantifying these features, their extremely short processing speed promises the ability to enable real-time characterization oriented toward high-volume cell cultures, as shown in Fig. 5c, where conventional computer vision algorithms show advantages in ultralarge-scale high-resolution imaging^78^.

### Point-of-care testing

**Fig. 6:**
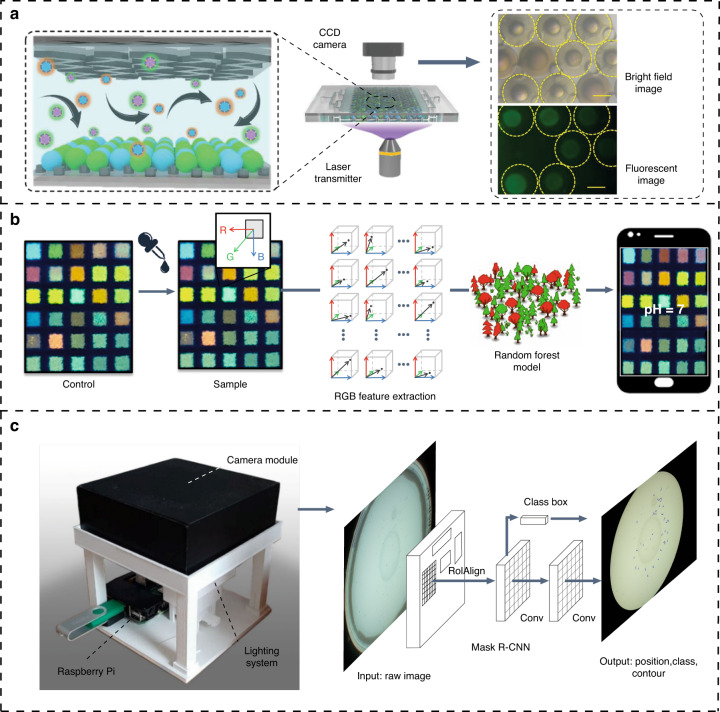
CV-assisted point-of-care testing devices. **a** Herringbone microfluidics for multiple exosome detection^84^. The detection of exosomes can be simplified to read the photonic signals of barcodes with the imaging of charge-coupled-device and the digital image processing methods to achieve higher sensitivity and accuracy. **b** A mobile device supported by machine learning for high-precision pH classification^88^. The color values from individual components are extracted by multiple traditional image processing techniques, such as gray/HSV conversion, histogram equalization and blurring, and calculating the difference in the vector space can improve the precision of pH classification. **c** A low-cost system for high-precision detection of *C. elegans*^96^. This system utilizes a Mask R-CNN to automatically detect *C. elegans* in an end-to-end manner, which yields reliable and precise location information. The neural network structure has been optimized and supported by hardware and software platforms, making it easy to deploy on mobile devices for a wide range of bioanalytical tasks

The global SARS-CoV-2 pandemic has led to a high demand for point-of-care testing (POCT) devices^79^. Microfluidic POCT platforms simplify analyses by integrating multiple procedures, such as sampling, chemical reactions, chromatographic separation and detection into a small chip, enabling multiple analyses and providing a “sample-in-answer-out” function^80,81^. Accurate and sensitive signal readout methods are essential for addressing analytical sensing, cost and operational difficulties and determining the practical applications of analytical sensing strategies, and the use of simplified but accurate readout methods is important for microfluidic instantaneous analytical sensing devices^82,83^. In Fig. 6a, Chen et al. ingeniously designed aptamer-functionalized barcodes and microfluidic chips. Various aptamers to multiple target exosomes can be encoded by the characteristic reflection peaks of barcodes, and the results of multiple detection of exosomes can be read by the photonic signals of the highly sensitive barcodes, which are represented as different brilliant structural colors under a camera^84^.

The availability of inexpensive imaging technology such as Raspberry Pi has lowered the barriers to accessing cost-efficient and objective detection methodologies, and they can be interfaced with flexible software that is capable of performing image segmentation and probing a variety of color spaces. However, this approach is unsuitable when the response is complex and irregular^85^. The computational capacity of integrated circuit chips has increased significantly, greatly enhancing digital signal processing and image processing capabilities. The lightweight trend in neural network research has led to the widespread deployment of computer vision models on mobile devices, such as the architectures of MobileNetV1 and MnasNet, which can also achieve faster speed and accuracy in digital signal detection and image processing. In the face of the demand for signal readout methods for POCT microfluidics, computer vision techniques can detect signal variations more sensitively, for example, by obtaining accurate pixel-by-pixel color characteristics and distinguishing subtle changes in color brightness and hue during a chromogenic reaction, thereby accurately quantifying the concentration of the target analyte and improving the accuracy of POCT^85–88^, as shown in Fig. 6b. Image processing algorithms can be integrated with lightweight convolutional neural networks on a model device to achieve fully automated real-time data readout, analysis and upload, even in remote areas with poor infrastructure^89^. On the other hand, the unique patterns and features extracted from the data by computer vision algorithms can reflect biomedical information that is difficult to obtain with traditional methods.

The built-in modules of smartphones enable them to collect and convert signals, analyze data, and transmit and store information. Image processing programs, software, and lightweight image detection models based on convolutional neural networks can be rapidly deployed on mobile devices, enabling automated image reconstruction and enhancement^90–92^ and quantitative analysis^82,93^ to carry out a wide range of cell analysis tasks, such as blood cell morphology analysis^94^, cell counting^95^ and cell tracking^96^, as shown in Fig. 6c. Image quality is limited by the parameters of the mobile phone imaging sensor, such as the field of view, resolution and other model parameters, and the shooting environment, leading to difficulties in capturing high-quality images in remote areas. The integration of noise reduction and resolution enhancement algorithms can help to improve image quality and thus improve POCT sensitivity.

There is no doubt that microfluidic POCT technology based on computer vision is a very promising area that enables the integration of a microfluidic chip with mobile devices for various cell detection needs. Traditional image processing algorithms can eliminate irrelevant information and restore useful information to enhance image realism for fast, high sensitivity colorimetric analysis and image preprocessing for object detection. Visual detection models based on neural networks, supported by transfer learning and cloud computing^92^, can significantly reduce the high memory requirements of POCT devices for the deployment of complex models. It can address a wide range of complex cellular analysis needs, greatly simplifying health care and improving clinical outcomes, especially in resource-constrained areas.

## Challenges and opportunities

The development of microfluidic technology in recent years has made it possible to reach a higher standard in cell imaging analysis. Computer vision, as an important technical means, can intuitively interpret the phenotypic characteristics and internal structure of each cell in a noninvasive or low-damage way to isolate, identify and analyze cells of interest in complex heterogeneous cell populations.

### Throughput vs. speed of imaging vs. image quality

Microfluidic systems have the potential to provide high-throughput analysis, rapid image acquisition, and high image quality, but achieving all three simultaneously can be challenging due to various technical limitations. One major limitation is the speed of imaging. High-speed imaging is essential for tracking the dynamic behaviors of cells in real time, such as cell migration, division, and response to external stimuli. However, the faster the imaging speed is, the lower the image quality, as the exposure time for each frame is reduced, which can result in motion blur, low contrast, and noise. Another limitation is the throughput of the microfluidic system. High-throughput analysis is essential for screening large cell populations or for analyzing multiple samples simultaneously. However, increasing the throughput typically requires sacrificing imaging speed or image quality, as the system needs to accommodate more cells or samples, which can lead to reduced imaging resolution or longer acquisition times. Finally, achieving high image quality can also be challenging due to limitations in optical resolution and sensitivity, as well as potential interference from the microfluidic device itself. This can result in lower image quality, particularly when imaging deep within a microchannel or through thick biological samples.

Thus, maintaining a balance among throughput, imaging speed, and image quality is necessary to achieve optimal performance in microfluidic cell analysis and to ensure that the data obtained are reliable and accurate. DeMello’s group presented a sheathless, microfluidic imaging flow cytometer incorporating stroboscopic illumination for blur-free cellular analysis at throughputs exceeding 50,000 cells/s^19,20^. Motion blur in images from fast-moving objects can be eliminated by appropriate stroboscopic light pulses. Stroboscopic illumination can boost the ambient brightness level in short pulses, which greatly reduces the exposure time of the camera. The increased brightness allows the whole system to run faster, and the higher light output also allows for a reduced aperture to provide a better depth of field for imaging. The single-cell flow formed by inertial focusing avoids the steps of object localization and segmentation in computer visual detection and speeds up the system image processing. In addition, approaches using multiple channels in parallel increase the detection throughput even further based on achieving a balance between high throughput, imaging speed and image quality. Holzner et al. presented an optofluidic flow cytometer integrating a refractive, microlens array, which could magnify micron-sized objects up to 4 times, not only providing optical performance equivalent to that of a high magnification microscope objective but also enhancing the throughput in wide field imaging and enumeration of cells^97^. Huang et al. adopted an image enhancement approach by using a generative adversarial network (GAN) model that enables the images acquired at low magnification to reach the resolution at high magnification, allowing the system to acquire high-quality images while maintaining high throughput at low resolution. These results have paved the way for the statistical analysis of cells with high-dimensional spatial information^98^.

Microfluidic flow cytometry achieves high cell detection throughput while delivering large amounts of high-quality cell image data. Relying solely on traditional image processing can easily result in the loss of information about heterogeneous cell populations; for instance, 1 mL of whole blood may contain only 1–10 circulating tumor cells. The development of deep learning algorithms has completely revolutionized the approach to image processing and the scale of image analysis^99^, transforming on-chip imaging into an intelligent engine capable of fully automated quantitative analysis that was previously unimaginable.

### Development of deep learning

With the rapid advancement of computer vision and microfluidic technology as well as improved hardware, complex cell visual analysis is attracting more attention in cell research. Artificial intelligence branches, supervised learning, unsupervised learning, reinforcement learning and transfer learning are being developed to significantly enhance computer vision neural networks in cell image analysis. Here, unsupervised learning or weakly supervised learning can extract information on the internal relationships of data without requiring domain experts to annotate the data^100^. It can also find hidden populations of cells that are rare or only slightly different from other cells that have not been observed; thus, it is useful for discovering new cell types or phenotypes^101–103^. Reinforcement learning is a process of interacting with the environment, receiving rewards or punishments after each action, learning through trial and error, and optimization with the goal of maximizing cumulative rewards. It has shown good results in omics analysis, biomedical image analysis, drug screening and other fields^104^ and performed satisfactorily in high-throughput microfluidics control, which has paved the way for robust and repeatable long-term microfluidics experiments^105,106^. On the other hand, applications of transfer learning can greatly improve the generalizability of neural networks, train the model faster, and obtain higher prediction accuracy^107^.

A major challenge in applying deep learning in microfluidics is the acquisition of training data. However, manually annotating datasets is time-consuming and highly dependent on the prior knowledge of the experts. Thus, there is uncertainty in the true value, that is, there are errors in the annotations provided by the experts. Active learning may be adopted to solve the problem of limited data labels and a high cost of data annotation and to avoid subjectivity and selection bias when experts annotate data. It was seen to improve the model generalizability^108^. Active learning can proactively propose some annotation requests, submit selected data to experts for annotation, and then train the model with the annotated data. The above process is repeated continuously until the model achieves the ideal accuracy^109^. In addition, the imbalance of data classes will affect the results of neural network analysis. In this case, data resampling and data enhancement are often used to balance the amount of data in each category, or the loss function is modified to add a larger penalty coefficient to the class with fewer samples so that the model training is more in favor of the class with fewer samples^110^.

In the development of microfluidic chips, multimodal data fusion is a future trend^111^. It integrates multiple detection methods into a chip to measure multiple parameters of the target, such as simultaneously measuring the size of a single cell, the polarizability under multiple frequencies and the deformability of cells in the process of cell tracking^112^ for more comprehensively characterized cells. Convolutional networks have proven to be effective in analyzing the impedance spectra generated by electrical impedance detection^113^, and the synergistic detection and cross-validation of optical imaging and electrical impedance is very promising^114^.

The combination of organ-chip and computer vision also has great potential to obtain microscopic cell deformation and movement that cannot be observed by the naked eye or through a microscope, such as cell migration in blood vessels^115^ and brain tumor metastasis in the blood‒brain barrier^116,117^. Chen et al. used an improved CNN to recognize and focus tumor spheroids automatically and to observe the structural changes and dynamic growth of tumors. It was found that CNNs could clearly extract cell features such as tumor spheroid boundaries and tumor surface roughness, accurately detect the degree of tumor spheroid invasion on 3D organ chips, and automatically generate analysis reports^118^. Computer vision techniques can also track the growth of cells in organs or organoids in real time, providing a more detailed, intuitive and comprehensive understanding of cell growth and movement^119^. They will play an important role in computer-assisted early cancer screening, tumor drug development and personalized medicine.

In summary, the analysis of cell composition, structure and morphology at the single-cell level is essential in various fields, such as cell biology, medical research, and drug discovery. In the past, manual observation of cell images was the primary way to extract information about cells. However, manual observation is time-consuming, prone to human errors and biases and is not practical when analyzing large datasets in the AI era. Therefore, computer vision techniques have emerged as an alternative to manual observation.

Traditional computer vision methods rely on feature extraction, which involves defining a set of handcrafted features based on the prior knowledge of researchers. These methods can perform cell feature extraction and some conversions regarding color and grayscale at a very fast speed. Thus, they have been widely used in high-throughput imaging cytometry. However, these methods are not suitable for analyzing complex cell images with a large number of features. When dealing with complex cellular images with a large number of features, deep learning neural networks have become increasingly popular.

As the examples in this review have indicated, deep learning neural networks can learn complex features directly from cell images without the need for feature extraction. These networks can learn and recognize cell structures and morphology and perform cell segmentation, cell recognition, and cell tracking tasks. In particular, CNN models can analyze large volumes of cell images with high accuracy and speed, which enables researchers to explore cell heterogeneity, reveal interactions among cells, and enhance the understanding of cellular processes.

## Conclusions

The combination of microfluidics and computer vision has led to significant advancements in cell analysis. In this review, microfluidic devices are shown to enable the manipulation and analysis of cells at the microscale level, providing high precision and throughput and generating specific cell patterns. On the other hand, computer vision algorithms can process large amounts of data, analyze the resulting images, extract quantitative data, and identify patterns that may not be visible to the human eye. The integration of these two technologies has allowed for more efficient and accurate cell analysis. This enables researchers to study cells in a highly controlled environment, generating high-quality data that can be used to answer complex research questions.

As computer vision algorithms continue to improve, we can expect to see even more advanced cell imaging analysis. For example, machine learning algorithms could be trained to recognize specific cell types or identify subtle changes in cell behavior. Additionally, the use of microfluidic devices combined with computer vision could enable high-throughput screening of drug candidates, allowing for faster and more efficient drug development.

In summary, the combination of microfluidics and computer vision has tremendous potential for advancing our understanding of cell behavior and developing new treatments for disease.
